# Detection of the myxosporean parasite *Parvicapsula pseudobranchicola* in Atlantic salmon (*Salmo salar* L.) using *in situ* hybridization (ISH)

**DOI:** 10.1186/s13071-015-0718-4

**Published:** 2015-02-15

**Authors:** Turhan Markussen, Celia Agusti, Egil Karlsbakk, Are Nylund, Øyvind Brevik, Haakon Hansen

**Affiliations:** Norwegian Veterinary Institute, P.O. Box 750 Sentrum, , N-0106 Oslo, Norway; Institute of Marine Research, P.O. Box 1870, , Nordnes, 5817 Bergen, Norway; Department of Biology, University of Bergen, Thormøhlensgt 55, N-5020 Bergen, Norway; Cermaq Group AS, Dronning Eufemias gate 16, N-0191 Oslo, Norway; Present address: Institute of Food Safety and Infection Biology, Norwegian University of Life Sciences, P.O. Box 8146, , Dep., NO-0033 Oslo, Norway

**Keywords:** *Parvicapsula pseudobranchicola*, Myxozoa, Atlantic salmon, Parasites, *in situ* hybridization, ISH, Real-time PCR, Locked nucleic acid probe, LNA probe

## Abstract

**Background:**

*Parvicapsula pseudobranchicola* is a marine myxosporean parasite infecting farmed Atlantic salmon (*Salmo salar*). A major site for the parasite is the pseudobranch, which may be destroyed in heavily infected fish. Parvicapsulosis may be associated with significant mortality, although the main effect of infections seems to be runting. *In situ* hybridization (ISH) is, in the absence of specific antibodies, the preferred method for the detection of cell- and tissue tropisms of myxozoans in the early phases of infection of the host, and provides information about the possible association between the pathogen and pathology. A positive diagnosis of parvicapsulosis is based on histopathology and PCR. The aim of the present work was to develop a specific, sensitive and robust ISH assay for the detection of *P. pseudobranchicola* in tissues.

**Methods:**

The ISH method was designed to specifically target *P. pseudobranchicola* 18S rDNA/rRNA using a locked nucleic acid (LNA) modified oligonucleotide probe. The method was tested on paraffin embedded *P. pseudobranchicola* infected pseudobranchs. The infections were confirmed by light microscopy revealing the presence of typical *P. pseudobranchicola* trophozoites and spores, and the presence of parasite was confirmed with real-time RT-PCR.

**Results:**

Specific regions stained by ISH overlapped well with the parasitized and degenerated regions in neighbouring HE stained sections. No staining was observed in pseudobranchs of Atlantic salmon which had been held in *P. pseudobranchicola*-free water.

**Conclusions:**

We report here the development of a sensitive ISH assay for the detection of *P. pseudobranchicola* in paraffin embedded tissue. The technique will be valuable in the study of host entry, early proliferation, pre-spore development, pathology and tissue tropism in Atlantic salmon.

## Background

Infections with the marine myxosporean *Parvicapsula pseudobranchicola* are common in farmed Atlantic salmon (*Salmo salar*) in Norway, especially in the northern regions [1-3]. The parasite mainly infects the pseudobranch, which may be swollen and covered with a whitish matter [2,4,5]. Affected fish often show eye bleeding, and may be anaemic, non-feeding and in poor condition. Since the blood supply to the eyes passes via the pseudobranchs, it has been suggested that massive infections by the parasite and tissue destruction may affect the blood supply and cause blindness [4,5]. In affected pseudobranchs, mature *P. pseudobranchicola* spores may be found interlamellary some 4-8 months after sea-transfer [1,4,5]. However, the time needed for the parasite to develop spores is poorly known since the timing of infection can only be estimated through screening using molecular methods, and the presence of spores in the population prior to the development of clinical parvicapsulosis has not been studied. In heavily infected pseudobranchs, there are few intact pseudobranch cells left [5]. However, the function of these cells, and consequently the organ itself, is not known [6]. Hence, the physiopathological effects of the infection are unknown. Foci of infection (spore development) have occasionally also been detected at other sites, such as in gills, kidney and liver [5].

Myxosporeans have two-host life cycles, with an alternate annelid worm host where fish-infective actinospores are produced. In the three currently known parvicapsulid life cycles, polychaetes from the order Sabellida are hosts [7-9], releasing actinospores. In the case of *P. pseudobranchicola*, both the annelid host and the early phases of infection in salmon are unknown. *In situ* hybridization (ISH) is the method of choice for the detection of myxozoans in early phases of fish infections, such as sporoplasm entry, migration and presporogonic proliferation in the fish tissues [10-12].

Screening with molecular methods have indicated that *P. pseudobranchicola* blood stages may occur transiently in infected salmon, and tissues with no known sporogeny have been found to contain parasite DNA [1]. Hence, the tissue and organ range exploited by the parasite is not well known, and may vary according to parasite development as in some other Myxosporea [13].

ISH is a technique that enables the direct detection of pathogen nucleic acids in histological sections. It may be difficult to link pathological findings to an agent, and here ISH has its strength compared to real-time PCR and traditional histopathology applied separately. The technique has previously been developed for several other parasites causing disease problems in aquaculture, including several myxozoans infecting salmonids e.g. *Tetracapsuloides bryosalmonae*, *Sphaerospora truttae*, *Ceratonova shasta* (formerly *Ceratomyxa shasta*)*, Myxobolus cerebralis* [10-12,14-16]. To be able to study early (pre-spore) stages of *P. pseudobranchicola*, detected in the past by molecular tools, we developed a specific and sensitive ISH method for the parasite. With this tool, described below, we may be able to pinpoint the port of entry, sites of early stages, follow parasite migration and reveal all affected cells and tissues.

## Methods

### Field samples

Pseudobranchs from an outbreak of parvicapsulosis at an Atlantic salmon (*Salmo salar* L.) farming site in Langsundet, Troms county in Northern Norway were collected from 100-500 g salmon in January 2013. Moribund fish were killed by a blow to the head. No approval from Institutional Animal Care and Use Committee (IACUC) or ethics committee was necessary. No experiments that involved fish were performed. The presence of *Parvicapsula pseudobranchicola*, was confirmed by histopathology and real-time RT-PCR (see below). The tissues were fixed (12 h) in buffered formalin and then transferred to 70% ethanol before being embedded in paraffin wax. Pseudobranchs from Atlantic salmon parr (20-40 g) kept in fresh water obtained from Hellefoss-Aamot hatchery (Buskerud county, Norway) were used as negative control.

### Real-time RT-PCR

Nucleic acids from ethanol fixed pseudobranchs were extracted using DNeasy Blood and Tissue kit, and from paraffin embedded sections using QIAamp DNA FFPE Tissue kit, following the protocol recommended by the manufacturer (Qiagen). As both protocols were run without RNase treatment, some RNA can be expected to be co-purified together with total DNA, according to the manufacturer. Real-time RT-PCR targeting *P. pseudobranchicola* 18S rDNA/rRNA was performed using 2 μl of DNA/RNA template and the Qiagen One-Step RT-PCR kit, following the protocol recommended by the manufacturer (Qiagen). A previously developed primer-probe set [17] was used, and the PCR was run on a Stratagene Mx3005P machine using the following cycling conditions; 50°C/30 min, 95°C/15 min followed by 40 cycles of 94°C/30 sec, 50°C/30 sec and 72°C/1 min and a final extension step at 72°C/10 min.

### In situ hybridization (ISH)

Standard precautions were made to ensure that all solutions and equipment used was nuclease free. The tissue sample producing the lowest Ct-value in the real-time RT-PCR was chosen for paraffin embedding, sectioned (3-5 μm) and mounted onto Superfrost™ Plus glass slides (Thermo Scientific). Sections were then incubated at 60°C for one hour, dewaxed in xylene 3 × 2 minutes and rehydrated in ethanol in decreasing concentrations (100%, 96%, 70% and 50%, 5 minutes each). After two quick washes and 2 × 5 minute incubation in PBS de Boer (PBS, pH7.4), endogenous enzymatic activity in the pseudobranch tissue slices were sufficiently neutralized by incubating in 0.1% H_2_O_2_ (Sigma-Aldrich) in methanol. Following 2 × 5 minute incubation in PBS, tissue sections were permeabilized with 40 μg/ml proteinase K (Novagen) in TE buffer (pH7.4) at 37°C for 15 minutes. The reaction was stopped by postfixation in freshly made 4% paraformaldehyde (Sigma-Aldrich) solubilized (w/v) in 0.1 M phosphate buffer (PB, pH7.4) for 6 minutes at 4°C. After 2 × 5 minute incubation in PBS, positive charges (that could lead to non-specific binding of probe) were neutralized by incubating the sections in 0.1 M triethanolamine buffer (TEA, pH8.0) containing 0.25% acetic anhydride (Sigma-Aldrich) for 5 minutes at room temperature. More acetic anhydride was added to a final concentration of 0.5% and the sections were incubated for another 5 minutes. After a 2 × 5 minute incubation in PBS, prehybridization was performed covering each section with 50 μl hybridization buffer. The hybridization buffer consisted of 4 × SSC (0.6 M NaCl, 60 mM sodium citrate, pH7.0), 0.2 g/ml dextran sulphate and 25 μl deionized formamide (both Sigma-Aldrich) mixed together and sonicated for 3-4 hours. The solution was then added polyA (250 μg/ml), ssDNA (250 μg/ml), tRNA (250 μg/ml), DDT (0.1 M) (all Sigma-Aldrich) and 0.5 × Denhardt’s solution (0.01% Ficoll 400, 0.01% polyvinylpyrrolidone and 0.01% bovine serum albumin, sterile filtrated). Hybridization buffer for general staining of all poly-A tailed mRNAs with polyT probe (see below) was identical to hybridization buffer except polyA was absent. Sections were incubated for 2 hours at 54°C followed by 5 minute incubation with 2 × SSC.

**Figure 1:**
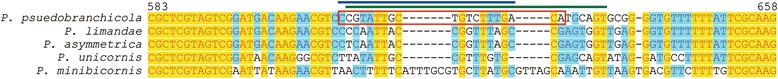
**Location of the** ***in situ*** **hybridization (ISH) probe sequence on the SSU rRNA gene of** ***Parvicapsula pseudobranchicola*** **in a partial multiple sequence alignment with other** ***Parvicapsula*** **spp. (** ***P. limandae*** **; EF429096,** ***P. asymmetrica;*** **AY584191,** ***P. unicornis;*** **AY584190 and** ***P. minibicornis;*** **AF201375).** Nucleotide positions are according to the partial *P. pseudobranchicola* sequence available in GenBank (AY308481). Red box = ISH probe target sequence. Green- and blue lines = Real-time PCR probe sequences from [3] and [17], respectively.

At present, only a 1663 nucleotide SSU rDNA sequence of *P. pseudobranchicola* is available in GenBank (AY308481). Therefore, an 18S rDNA/rRNAregion, the most variable gene region when comparing with the other closely related *Parvicapusla spp.*, was targeted in ISH probe design. The targeted region overlaps with that of previously developed real-time PCR probes designed for this parasite [3,17] (Figure 1). A locked nucleic acid (LNA) type modified probe (LNA nucleotides are DNA nucleotide analogues) was chosen as they have shown to be superior to classical non-modified DNA probes in several studies [18-20]. The final LNA probe (i.e. a DNA probe containing several LNA residues) Parvi_LNA probe: 5’-TGTCAAAGACAGCAATACGG-3’ was designed using the tool available at https://www.exiqon.com/mRNA-probes. The probe was designed to target both 18S rDNA and 18S rRNA. The calculated melting temperature (Tm) of the probe is 84°C (RNA) and 75°C (DNA) according to the manufacturer (Exiqon). BLAST searches using the probe sequence as query were made to ensure minimal risk of it binding to Atlantic salmon genome sequences. A LNA-modified polyT probe, PolyT(25)Vn, ordered from the same manufacturer, was also included to enable evaluation of the integrity of all polyA-tailed mRNA in the sections. Both LNA probes were ordered digoxigenin (DIG)-labeled in both ends. The concentration of each probe in hybridization buffer was optimized to 500 nM, a volume of 50 μl was added to the sections and hybridization was performed overnight at 54°C. Post-hybridization washes were carried out using 1 × SSC/10 mM DDT (2×) and 0.5 × SSC/10 mM DDT (3×), incubating for 15 minutes at 60°C for the first four washes and 10 minutes at room temperature for the final wash. The sections were then incubated 3 × 5 minutes in PBS and blocked for 30 minutes at room temperature using the blocking solution from the Tyramide Signal Amplification (TSA) kit (Perkin Elmer (see below)). A volume of 50 μl anti-DIG HRP antibody (Abcam) (1:400) in blocking solution was added to each section followed by an overnight incubation at 4°C. The next day sections were incubated 3 × 5 minutes in PBS and stained with the Biotin Tyramide, Streptavidin-HRP (1:100) (both from TSA kit) and 3-amino-9-ethylcarbazole (AEC) (Dako). Sections were incubated with AEC for 10 minutes (Parvi_LNA probe) or 2-4 minutes (PolyT(25)Vn). Washing between steps was performed with PBS (3 × 5 minutes each time). Otherwise, staining was performed following the recommendations by the manufacturers. Counterstaining was performed with Mayer’s hematoxylin (40 seconds) and slides mounted using Aquatex mounting agent (Merck). Morphology visualization for sections not undergoing the ISH procedure was performed with hematoxylin and eosin (HE). Light microscopy was conducted using a Leica DM5000B microscope and photos taken using an attached Nikon DXM digital camera.

## Results

Histology on pseudobranch samples from the disease outbreak revealed large numbers of myxosporean trophozoites and spores with morphological characteristics consistent with *Parvicapsula pseudobranchicola* [4,5]. Real-time RT-PCR verified the presence of *P. pseudobranchicola*, producing Ct-values averaging 19. Similarly, real-time RT-PCR on a nucleic acid extract from one paraffin embedded section neighbouring those used in ISH produced a Ct-value of 21.

**Figure 2:**
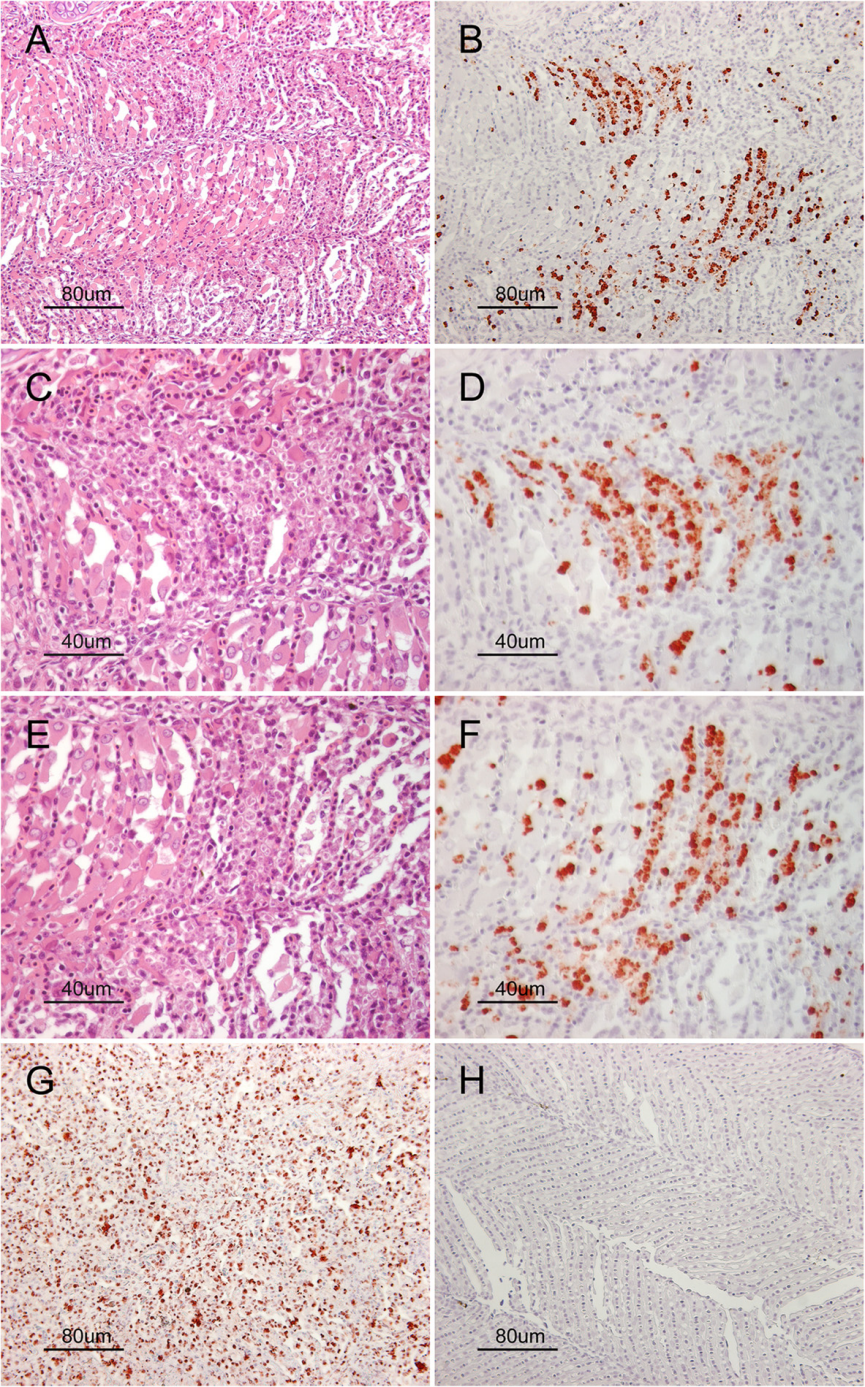
***In situ*** **hybridization (ISH) on** ***Parvicapsula pseudobranchicola*** **positive pseudobranchs from Atlantic salmon. A)** Hematoxylin and Eosin stained tissue section covering both healthy and degenerate regions in the tissue and **B)** the result from *in situ* hybridization using the *P. pseudobranchicola* specific LNA probe on a neighbouring section of the same sample, showing specific staining in the same tissue region (20x magnification). **C)** and **E)** are enlarged sections from **A)** (40x magnification) with the corresponding results from ISH shown in **D)** and **F)**, respectively. ISH with the LNA-modified polyT probe on a section from the same sample is shown in **G)**, and **H)** shows ISH result in *P. pseudobranchicola* negative pseudobranch.

The results from ISH on sections of paraffin embedded Atlantic salmon pseudobranchs positive for *P. pseudobranchicola* are shown in Figure 2. The top two photos show a hematoxylin and eosin (HE) stained section (A) and the result from ISH using the *P. pseudobranchicola* probe on a neighbouring section (B). The following two pairs of photos (C, D and E, F) are enlarged sections of the top pair of photos in the figure. In the examined material, clearly ISH stained parasite stages were observed interlamellary in the pseudobranch in regions with few intact pseudobranch cells. Staining was not seen intralamellary. The staining is specific for the parasite stages packed in degenerated regions, as also verified in the corresponding HE stained sections. The use of the polyT probe, which binds all polyA-tailed mRNAs, strongly suggest that the RNA had not been significantly degraded during the ISH procedure (Figure 2G). The *P. pseudobranchicola* probe does not bind genome sequences in Atlantic salmon, as no staining could be observed from *P. pseudobranchicola*-free fresh water kept Atlantic salmon pseudobranchs (Figure 2H).

## Discussion

The early development of *P. pseudobranchicola* in Atlantic salmon is not known, but a presporogonic proliferation and development seems to be present since non-target organs and tissues may be PCR positive after sea transfer prior to the appearance of spores in pseudobranchs [1]. To study the sites and pre-spore development of *P. pseudobranchicola*, we have in the present work developed a sensitive *in situ* hybridization (ISH) method for its detection in tissue sections.

*In situ* detection methodology, such as ISH, enables a direct link between the pathogen and cellular pathology in the tissue. ISH can detect low levels of parasite and, as the method targets the DNA/RNA of the pathogen, it can identify a specific myxosporean parasite regardless of its stage [11]. ISH therefore allows an identification of myxozoans in the host tissues, prior to the appearance of spores at their target site [11,12,15,21]. An ISH assay has previously been developed for *P. minibicornis*, and was used to detect this parasite in sections of PCR positive material lacking spores [22]. Previous observations on *P. pseudobranchicola* tropism were based on conventional PCR, and hence only revealed presence or absence of parasite DNA [1].

At present, only genomic small subunit rDNA (SSU rDNA) sequences are available in GenBank for *P. pseudobranchicola*. Hence the ISH probe design targeted the most variable region of the 18SrDNA/rRNA in order to minimize the possibility of cross hybridization to closely related *Parvicapsula* spp (Figure 1). However, in European waters, *P. pseudobranchicola* is the only parvicapsulid known to infect salmonids. Material was not available for the testing of the assay on closely related *Parvicapsula* spp. such as *P. asymmetrica* in lumpsucker (*Cyclopterus lumpus*) or *P. limandae* in dab (*Limanda limanda*), both parasites infecting the kidneys of their hosts. However, the 20 nt long LNA modified oligonucleotide probe displays low sequence identity (≤55%) towards the most closely related species (i.e. reverse complement sequence); *P. limandae* (11/20 nt), *P. asymmetrica* (10/20 nt) and *P. unicornis* (10/20 nt, including a one nt gap) (Figure 1). The potential for cross hybridization to these species by the probe should therefore be negligible. Towards other *Parvicapsula* spp., sequence identity is much lower. Also, although direct comparison between the two methods should be made with caution, it is relevant that a previously published diagnostic real-time PCR probe, having a sequence that largely overlaps with the ISH probe sequence, does not cross react with the listed most closely related *Parvicapsula* spp. [3]. The SSU rDNA region chosen for our ISH probe design is likely to be the best sequence region to target when designing short LNA-modified probes for the related *Parvicapsula* species. The presence of several LNA residues in an ISH probe enhances hybridization efficiency and specificity significantly compared to longer probes [18-20,23], and a short ISH probe can also be expected to exhibit better tissue penetration ability. We tested the developed ISH assay on salmon pseudobranch sections known to be infected with *P. pseudobranchicola*, but there is no reason why it should not work equally well on other organs or hosts (e.g. polychaete alternate hosts or other fish hosts) if the parasite is present.

The results from ISH showed specific regions of staining overlapping well with the parasitized and degenerated regions in the corresponding HE stained sections. Unspecific staining in the sections was minimal, especially when TSA amplification was applied. Without amplification a low level of background staining could be observed (not shown). No staining occurred in tissue sections from *P. pseudobranchicola*-negative fish. Hence, the probe does not bind genome sequences or RNA in Atlantic salmon. The probe was designed to target both rDNA and rRNA for highest possible sensitivity and the ISH assay was repeated three times with similar results (not shown). The ISH results from using the LNA-modified polyT probe, which binds polyA-tailed mRNAs, indicated that the RNA in the studied tissues had not undergone any significant degradation. An LNA modified probe with a reverse complementary sequence to that of the probe used (to investigate specific binding only to rDNA) was not tested as the aim of the present study was highest possible sensitivity. An ISH probe that targets both rDNA and its rRNA product can be assumed to be better for this purpose.

## Conclusion

We have developed a sensitive locked nucleic acid (LNA)-based *in situ* hybridization (ISH) assay for the detection of *P. pseudobranchicola* in paraffin embedded tissue. The technique presented herein is currently being applied on a set of tissue samples from Atlantic salmon smolts taken at regular intervals after sea-transfer. This work, combined with quantitative PCR analyses, should provide insight into tissue tropism, the presporogonic pathways of the parasite, and thereby disease development.
